# *QuickStats:* Percentage* of Adults Aged ≥18 Years Who Did Not Take Their Medication as Prescribed or Asked for Lower-Cost Medication to Save Money Among Those Prescribed Medication in the Past 12 Months,**^†^** by Number of Chronic Conditions**^§^** — National Health Interview Survey, 2018**^¶^**

**DOI:** 10.15585/mmwr.mm6843a6

**Published:** 2019-11-01

**Figure:**
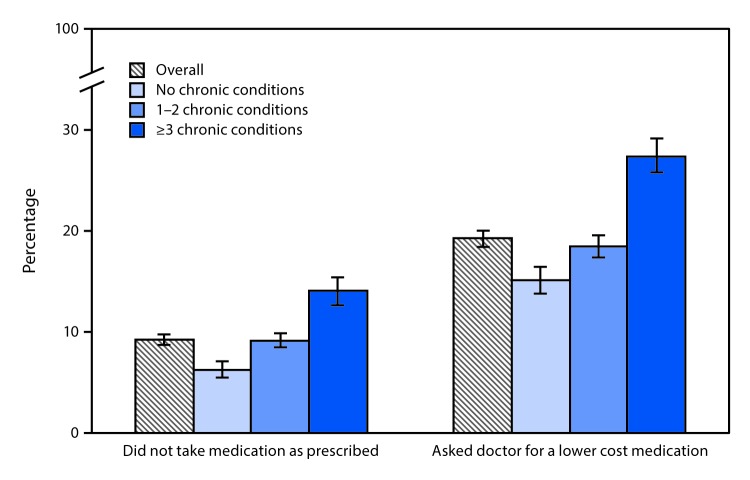
In 2018, among adults aged ≥18 years who were prescribed medication in the past 12 months, the percentage who did not take their medication as prescribed to save money increased with the number of reported chronic conditions, from 6.2% with no chronic conditions to 9.1% with 1–2 chronic conditions and 14.0% with ≥3 chronic conditions. The percentage who asked their doctor for a lower-cost medication also increased with the number of reported chronic conditions from 15.1% among those with no chronic conditions to 18.4% among those with 1–2 chronic conditions and 27.4% among those with ≥3 chronic conditions.

**Source:** National Health Interview Survey, 2018 data. https://www.cdc.gov/nchs/nhis.htm.

